# Estimating cost-effectiveness associated with all-oral regimen for chronic hepatitis C in China

**DOI:** 10.1371/journal.pone.0175189

**Published:** 2017-04-05

**Authors:** Hai Chen, Lijun Chen

**Affiliations:** Department of Infectious Diseases Control, Wuxi Center for Disease Control and Prevention, Wuxi, Jiangsu, China; Kaohsiung Medical University Chung Ho Memorial Hospital, TAIWAN

## Abstract

**Background:**

All-oral regimens are associated with higher effectiveness and shorter treatment duration for chronic hepatitis C. Given its superior effect and enormous patients in China, clinicians or patients may be compelled to consider delaying treatment for all-oral regimen.

**Objective:**

To estimate cost-effectiveness of delaying treatment for all-oral regimen in the subsequent years under different assumptions about their price and efficacy compared with standard of care in China.

**Methods:**

A state-transition Markov model was developed to estimate lifetime costs and quality-adjusted life years (QALYs). Incremental cost-effectiveness ratio (ICER) and net monetary benefit (NB) were calculated. And sensitivity analyses were also performed to assess the impact of uncertainty.

**Results:**

For treatment naive patients with Genotype 1, immediate treatment with all-oral regimen under assumed cost and efficacy at present was cost-effective compared with peginterferon α-2a (PegIFN) regimen at present with an ICER of $12536 per QALY gained and a positive NB of $6832 at a willingness-to-pay threshold of $21209. And it was more than 95% likely to be cost-effective if weekly drug cost was less than $1000. Moreover, patients delaying treatment for all-oral regimen in the 1^st^ year were associated with increase in QALYs of 0.62 and increase in cost of $10114 compared with initiating PegIFN regimen at present, which resulted in a positive NB of $3115.

**Conclusion:**

From a payer perspective, all-oral regimen is associated with good long-term health and economic benefit for treatment-naive patients infected with HCV genotype 1. Particularly, if all-oral regimen would become available at lower price in the future, delaying treatment for all-oral regimen may be a good choice for patients in China.

## Introduction

Hepatitis C virus (HCV) infection is a growing public health problem in both developing and developed countries [1]. Recent study [2] indicates that there are approximate 25 million patients infected by HCV in China which represent 1.8–3.7% of the total Chinese population. Among the infected patients [3], 25% of them have viral clearance while the remaining 75% may progress to chronic infection after 6 months of infection. Patients with chronic Hepatitis C (CHC) are at risk of undergoing fibrosis progression and developing advanced liver-related complications such as decompensated cirrhosis, hepatocellular carcinoma and liver-related death, resulting in substantial economic and health burden for both patients and society [4].

Until then, the standard of care in China for treatment naïve patients infected with genotype 1 is peginterferon α-2a (PegIFN) or interferon α (IFN) plus ribavirin (RBV) for 48 weeks [5]. These regimens have potential side effects and tedious treatment course, leading to poor tolerability profile especially in cirrhosis patients. With the advent of all-oral regimens, HCV treatment can for the first time be provided without interferon-based therapy, which is associated with considerable toxicity. As a result, previous suboptimal experience may be avoided.

All-oral regimens may represent important improvements over current options. Ledipasvir/sofosbuvir (Harvoni, Gilead Sciences), ombitasvir/paritaprevir/ritonavir/dasabuvir (Viekira XR, AbbVie) and elbasvir/grazoprevir (Zepatier, Merck) could achieve SVR rate >90% and had acceptable safety profile with shorter treatment duration [6]. In China, phase Ⅲ clinical trial of the first all-oral regimen (Daclatasvir and Asunaprevir) for hepatitis C in China by Bristol-Myers was successfully completed. But it has not been registered or approved by State food and drug administration (CFDA). It may take some time to go into clinical use. Although these new regimens have not been available in China at present, the rapid development of treatment for HCV represents a new hope for Chinese patients and the possibility of these regimens entering into China always exists because of the superior effect and enormous patients in China. In addition, the updated Chinese guidelines of prevention and treatment for CHC have been approved recently [5]. According to the guideline, immediate treatment of direct acting antivirals (DAAs) is recommended if patients could purchase from abroad and afford medical expenses during the course of treatment or waiting for them being used clinically in China.

Little is known about the relative economic and health benefit of all-oral regimens for hepatitis C compared with current options in China. Therefore, the aim of our analysis was, first, to explore the balance between health benefit and expenditures under different assumptions about the price and efficacy, and second, to estimate cost-effectiveness of delaying treatment for all-oral regimens in the subsequent years given that these regimens will soon be available in China.

## Materials and methods

### Model structure and assumptions

**Fig 1:**
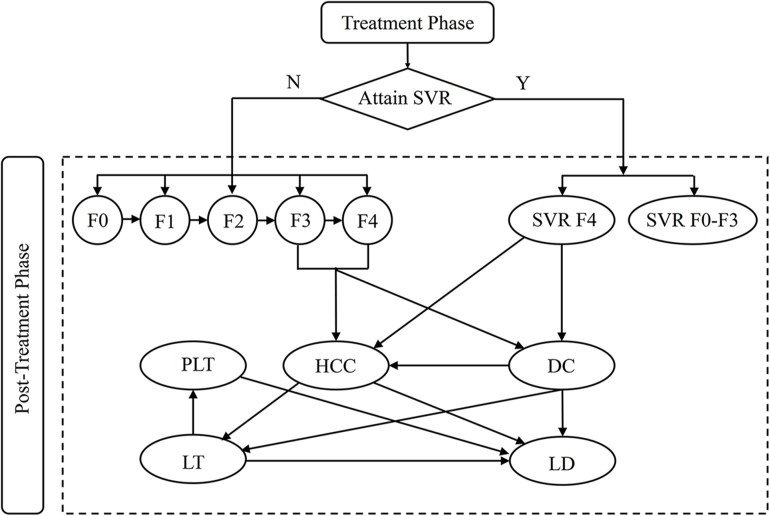
Markov model of disease progression. SVR, sustained virologic response; F0-F4, Metavir fibrosis scores; DC, decompensated cirrhosis; HCC, hepatocellular carcinoma; LT, liver transplant; PLT, post-liver transplant; LD, liver-related death. SVR F4, patients diagnosed at F4 achieved SVR; SVR F0-F3, patients diagnosed at F0-F3 achieved SVR.

Because CHC is a chronic disease characterized by the ongoing risk of fibrosis progression and developing advanced liver-related complications over time, a Markov modeling technique is appropriate [7]. In this study, a state-transition Markov model was developed in Microsoft Excel 2013 software (Microsoft, Redmond, WA) to estimate long term costs and quality-adjusted life years (QALYs) associated with different treatment regimens. Annual cycles and lifetime horizon were adopted. The detailed model is shown in Fig 1. First, patients entered at the initiation of treatment may receive either all-oral regimen or PegIFN regimen at different stages of liver fibrosis (METAVIR fibrosis scores: F0 no fibrosis; F1 portal fibrosis without septa; F2 portal fibrosis with few septa; F3 numerous septa without cirrhosis and F4 cirrhosis). After completion of treatment, a state-transition markov model was used to simulate lifetime disease progression. Patients who achieved SVR with F0-F3 were assumed to maintain SVR and to experience no further advanced disease progression until their death [8]. Instead, patients with cirrhosis (F4) continued to be at risk for developing liver-related complications even if they achieved SVR. But the probability was less than that of untreated patients with cirrhosis [9]. In addition, patients who failed to achieve an SVR or did not receive treatment returned to fibrosis progression or developing decompensated cirrhosis and hepatocellular carcinoma [10]. Those with decompensated cirrhosis or hepatocellular carcinoma could receive liver transplantation. Patients with advanced liver-related complications had excess risk of death. Furthermore, age and sex specific other-cause mortality based on Chinese population was applied to each health state.

### Patient population

Approximately 56.8% of HCV-positive patients were infected with HCV genotype 1b in China [11]. Most of the patients had lower public awareness of hepatitis C and also remained untreated [2]. And due to the limited data and time, the target patient population of this analysis only consisted of treatment naïve patients infected with HCV genotype 1 in China. Our model followed a hypothetical cohort of 1000 patients. And characteristics of baseline such as mean age, sex and fibrosis stages of the simulated patient population were based on a recent Chinese study [12]. Enrolled patients had a mean of 50 years of age and 52.0% of them were male. The baseline distribution of fibrosis stages was listed as follows: F0 0.8%, F1 45.5%, F2 41.3%, F3 9.9% and F4 2.5%. As time goes on, ages and fibrosis stages of different patients were constantly updated.

### Treatment regimens and clinical inputs

According to Chinese guideline [5], all-oral treatment regimen for 12 weeks with or without RBV was recommended for treatment naïve patients with or without compensated cirrhosis. As a reference case, PegIFN plus RBV for 48 weeks (assumed to be 6% of patients discontinued due to standard futility rule) was served as comparator regimen [13]. Patients with decrease of HCV RNA<2log10 IU/mL at week 12 discontinued treatment as a standard futility rule. The primary efficacy measure in the model was SVR rates, which were assessed 12 weeks after the end of treatment [14]. Due to lack of treatment efficacy based on Chinese population, SVR rates of all-oral regimen was assumed with a wide range from 85% to 95%. And according to clinical trials and meta-analysis in China, SVR rates of PegIFN plus RBV for 48 weeks were 70% for F0-F3 and 65% for F4 [15, 16].

### Transition probability

According to the natural history of CHC, patients without cirrhosis could transition to a more advanced stage of fibrosis [17]. Because well-designed, long-term prospective studies in patients with CHC in China are lacking, the estimates from Canada were used which were based on a meta-analysis using Markov maximum likelihood estimation [18]. Patients with F3 or F4 could additionally progress to decompensated cirrhosis or hepatocellular carcinoma [19, 20]. The probability of those with decompensated cirrhosis transition to hepatocellular carcinoma was obtained from a study by Planas et al [21]. And the probabilities of liver transplant from decompensated cirrhosis or hepatocellular carcinoma were obtained from Davis GL et al [22]. Particularly, the excess mortality rates with advanced complications were based on Chinese studies [23–25]. In addition, the mortality rate of liver-transplant was estimated from the liver transplantation society of Japan [26]. Patients with cirrhosis who achieved SVR could still experience advanced complications, but at a reduced rate compared with those not achieving SVR (*RR* = 0.0857 and 0.2400 for decompensated cirrhosis and hepatocellular carcinoma, respectively) [19]. Moreover, all states could lead to natural death, in accordance with the gender and age-specific other-cause mortality rates from the National Bureau of Statistics of China in 2010 [27]. Annual transition probabilities are presented in S1 Table.

### Cost inputs

Only direct medical cost was included from the payer perspective. And there were three types of cost: drug costs, monitoring costs and health state costs. For each health state, in-patient, out-patient, emergency department, ambulatory and pharmacy costs were accounted for. All costs were converted to USD using official exchange rates as of 2016 (1 USD = 6.6594 CNY) and were inflated to 2016 prices using China Consumer Price Index (CPI) [28]. Since all-oral regimen has not been available in China market, its pricing is still unknown and it could change as new drugs enter the market. Therefore, weekly drug cost of all-oral regimen was assumed with a wide range from $1000 to $3000. At present, patients with HCV in China are mainly treated with PegIFN 180μg/0.5ml per week in combination with RBV (1000 mg per day) [29]. According to experts’ option and charging standard of hospital, the weekly cost of PegIFN and RBV were $171 and $3, respectively. Moreover, weekly cost of monitoring was $25, with the assumption that this cost was identical for each treatment regimen. The costs associated with the management of adverse events were not included in the analysis as all-oral regimen had fewer side effects than interferon-based treatment and they were anticipated to have minimal impact.

The average costs applied to each health state were derived from published literature. A cross sectional survey had reported that more advanced the disease progress, higher costs to the condition [30]. For patients at fibrosis stage, the medium cost was $1292; for patients of cirrhosis, the medium cost was $1338. But this study only reported inpatient costs. It was not appropriate for advanced complications because of more than once for hospitalization and excess costs from outpatient. So annual costs of decompensated cirrhosis and hepatocellular carcinoma were estimated from studies on chronic hepatitis B (CHB) in China [31, 32], with the assumption made specifically for this study: annual costs for advanced complications of CHB patients are identical to CHC patients. Furthermore, the estimated annual costs for patients receiving liver transplant were derived from Chinese patients [33, 34]. And patients who achieved SVR were assumed not to incur any further HCV-related medical costs. Future costs were discounted at 3% per year. All the costs are presented in S2 Table.

### Utility inputs

An alternative approach to health-related quality of life (HRQOL) involves the measurement of utilities, to indicate preferences for particular health outcomes [35]. Health-state utilities are global measurements of quality of life on a scale from 0 (death) to 1 (full health) and allows both morbidity and mortality to be combined into a single weighted measure such as QALY [36]. Utility decrements were assigned to each health state of patients who did not achieve SVR or remained treated. There are limited data on the utilities of health states for CHC in China. So the utilities denoted in this study were mainly taken from the literature published by McLernon et al [37]. Patients who achieved SVR were assumed to receive a utility increment of 0.05 with living in the fibrosis stages [38]. And the utility decrement of 14.6% was assigned to PegIFN regimen because of the negative impact on quality of life associated with adverse events. However, in account of safety profile and good tolerance of all-oral regimen, a utility increment of 4.5% was applied [39]. Future QALYs were discounted at 3% per year. All the utilities are presented in S3 Table.

### Model consistency

Assessing the consistency of the model was classified into three categories: internal consistency, external consistency, and between-model consistency [40]. To investigate the internal consistency, sensitivity analyses were carried out including deterministic and probabilistic sensitivity analysis. And external consistency was investigated by comparing incidence of advanced liver-related complications with the rates estimated on the basis of epidemiologic studies in China. According to a cohort study in Chinese commercial plasma donors [41], the incidence of liver cirrhosis was 10.03%, hepatocellular carcinoma was 2.90% and liver-related death was 3.90%. No antiviral treatment was applied during the period of infection. After the markov simulation of natural history, we gained the incidence of liver cirrhosis was 10.10%, hepatocellular carcinoma was 2.80% and liver related death was 2.70% under our base-case assumptions. The results were identical to those from the cohort study.

What’s more, between-model consistency was also assessed by comparing some features of our model with those of existing models and by comparing results simulated from our model with those of Ferrante et al [42]. After the simulation, validating to lifetime costs and QALYs produced *R*^*2*^ statistics of 0.979, 0.997, respectively (S1 Fig). And comparing to the reported costs and QALYs also showed no significant difference (*P* = 0.128 and *P* = 0.161, respectively).

### Model analysis

**Fig 2:**
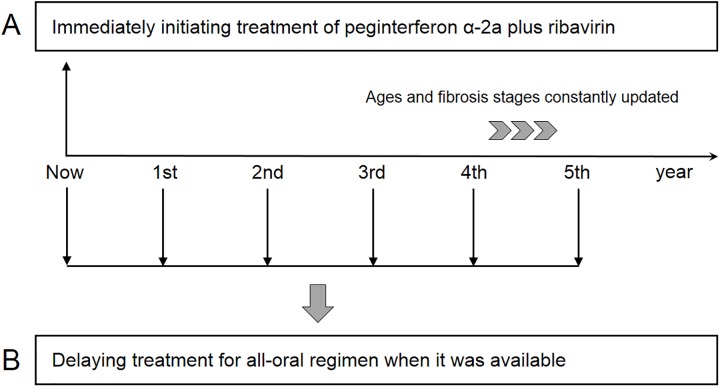
Scenario analysis under assumed SVR rate and weekly drug cost. Scenarios including (A) immediate treatment with peginterferon α-2a (PegIFN) regimen and (B) delaying treatment for upcoming all-oral regimen when it was available in the next five years. Ages and fibrosis stages constantly updated.

When considering options, several factors might affect the decision to adopt appropriate treatment regimen. Most of patients may suffer from side effects because of IFN-based regimen (PegIFN). Balancing the risk of side effects and treatment effectiveness, delaying treatment for upcoming all-oral regimen would be a considerable approach for them. Therefore, scenario analysis was conducted to evaluate potential cost-effectiveness under assumed price and efficacy for different scenarios including (a) Immediate treatment with PegIFN regimen (b) delaying treatment for upcoming all-oral regimen when it was available in the next five years (Fig 2). Incremental cost-effectiveness ratio (ICER) and net monetary benefit (NB) were calculated in order to quantify economic benefits.

$$ICER=\frac{C_{1}-C_{2}}{E_{1}-E_{2}}=\frac{\Delta C}{\Delta E}$$ (1)

$$NB=\lambda \times \left(E_{1}-E_{2}\right)-\left(C_{1}-C_{2}\right)=\lambda \times \Delta E-\Delta C$$ (2)

*C*_1_ is the total cost of comparative regimen (higher), *C*_2_ is the total cost of reference treatment regimen, *E*_1_ is the total effectiveness of comparative regimen (higher), *E*_2_ is the total effectiveness of reference regimen and λ is the cost-effective threshold (willingness-to-pay). Lifetime costs and quality-adjusted life years (QALYs) associated with different treatment regimens were estimated from markov model. According to suggestions from World Health Organization (WHO), three times of GDP per capita worked as the cost-effective threshold. Gross domestic product (GDP) per capita of 2014 was $7070 (1 USD = 6.6594 CNY) based on the national data from National Bureau of Statistics of China [43]. Therefore, to ascertain the cost-effectiveness, we used a cutoff of $21209 in this study.

Moreover, baseline characteristic (mean age or fibrosis stage) also has an impact on cost-effectiveness in the next five years. Patients should understand their fibrosis stage to help guide their own treatment decisions at different ages. And to accommodate uncertainty of cost-effectiveness results, one-way sensitivity analysis was conducted with fixed weekly drug cost of $2000 and SVR rate of 90%. Key inputs including transition probabilities (95% CI), utility estimates (±20%), discounting rate (±3%), SVR rates (±20% to a maximum of 100%) and weekly drug cost (±20%) were varied in the way that was similar to the recent study [44]. Particularly, because all-oral regimen is not available in China at present. Therefore, it is necessary to estimate impact on different assumptions about cost and SVR rate.

Moreover, probabilistic sensitivity analysis was conducted and all the inputs of model were varied simultaneously. Ranges of different inputs were used to calculate the standard error for each input, which was then utilized to determine the key parameters of each specific distribution. Beta distribution was assigned to transition probabilities, utility estimates and SVR rates; Gamma distribution was assigned to each cost. 1000 independent sets of inputs were sampled from the probability distributions and a cohort of 1000 hypothetical patients per treatment regimen was modeled for each set. The results were reported using cost-effectiveness acceptability curves.

## Results

### Scenario analysis

Lifetime cumulative costs and QALYs of each regimen were estimated in Table 1. Immediate treatment of all-oral regimen at present was associated with overall increase in QALYs of 0.79 and increase in cost of $9876 compared with PegIFN regimen, which was a cost-effective option with an ICER of $12536 per QALY gained and a positive NB of $6832. As time goes on, for treatment-naive patients, delaying treatment for all-oral regimen in the next five years from now on were also estimated. If patients initiated all-oral regimen in the 1^st^ (2^nd^, 3^rd^) year, this option would result in a 54% (120%, 201%) reduction of NB compared with those treated immediately at present. Patients initiating all-oral regimen in the 1^st^ year were associated with overall increase in QALYs of 0.62 and increase in cost of $10114 compared with PegIFN regimen, which resulted in a positive NB of $3115 and those in the next years led to negative NBs. In conclusion, patients could wait for up to one year until all-oral regimen became available. Otherwise, patients should be treated with PegIFN regimen at present. The earlier patients initiating all-oral regimen, the more economic benefits will be gained.

**Table 1:** Cost-effectiveness of all-oral regimen versus PegIFN regimen in the next five years.

		Total		Incremental		ICER($/QALY)	NB($)
		QALY	Cost($)	QALY	Cost($)		
PegIFN regimen		15.15	16209	—			
All-oral regimen	Now	15.94	26085	0.79	9876	12536	6832 (Ref.)
	1^st^ year	15.78	26322	0.62	10114	16215	3115 (-54%)
	2^nd^ year	15.57	26492	0.42	10284	24529	-1392 (-120%)
	3^rd^ year	15.32	26594	0.16	10385	63169	-6899 (-201%)
	5^th^ year	14.68	26599	-0.47	10390	-22099	-20362 (-398%)
	4^th^ year	15.02	26629	-0.13	10420	-77912	-13257 (-294%)

PegIFN regimen, Peginterferon α-2a plus Ribavirin; QALY, quality-adjusted life year; ICER, Incremental cost-effectiveness ratio; NB, net monetary benefit.

### Subgroup analysis

**Fig 3:**
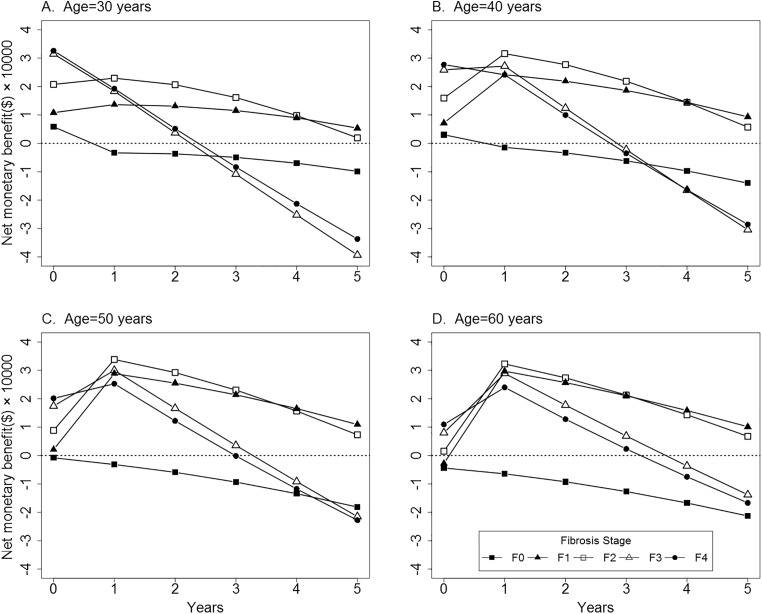
Cost-effectiveness of delaying treatment for all-oral regimen under different combinations of age and fibrosis stage in the next five years. Mean age of patients diagnosed at F0-F4 ranged from 30 years to 60 years and net monetary benefit was calculated. F0-F4, Metavir fibrosis scores.

According to baseline characteristic, estimates of cost-effectiveness were stratified by a series of 20 cohorts which represent possible combinations of age and fibrosis stage (4 × 5 = 20) in Fig 3. Patients diagnosed at F0 fibrosis achieved the negative NB of initiating all-oral regimen in the next five years. If they could not assess all-oral regimen at present, immediate treatment of PegIFN regimen was recommended. On the contrary, for those diagnosed at F1 or F2 fibrosis, delaying treatment for all-oral regimen appeared to be cost-effective in the next five years compared with those initiating PegIFN regimen at present. In addition, patients with F3 or F4 fibrosis delaying treatment for all-oral regimen appeared to be cost-effective in the next two years.

### Sensitivity analysis

The result of base-case was sensitive to variations in utility estimates, weekly drug cost and SVR rates. One-way sensitivity analysis was presented using tornado diagrams in S2 Fig. And because the pricing and efficacy of all-oral regimen have not been determined, a variation of weekly drug cost from $1000 to $3000 was applied under three levels of SVR rates (85%, 90% and 95%). ICERs varied significantly from -$892 to $36537/QALY. All-oral regimen with higher SVR rate and lower cost were associated with lower ICER. For example, if all-oral regimen could achieve a SVR rate of 90%, it would remain cost-effective even if weekly drug cost increased to $2500 (Table 2). And all-oral regimen would be cost-saving if weekly drug cost was less than $1000.

**Table 2:** Cost-effectiveness of all-oral regimen versus PegIFN regimen at present under different assumption of SVR rate and weekly drug cost.

SVR(%)	Weekly drug cost($)	Total		Incremental		ICER($/QALY)	NB($)	GDP per capita	
		QALY	Cost($)	QALY	Cost($)			<1×	<3×
85	1000	15.78	15653	0.62	-555	-892	13758	***	***
	1500		21479		5270	8466	7933	**×**	**√**
	2000		27304		11095	17823	2108	**×**	**√**
	2500		33129		16921	27180	-3717	**×**	**×**
	3000		38954		22746	36537	-9543	**×**	**×**
90	1000	15.94	14434	0.79	-1774	-2252	18483	***	***
	1500		20259		4051	5142	12656	**√**	**√**
	2000		26085		9876	12536	6832	**×**	**√**
	2500		31910		15701	19931	1007	**×**	**√**
	3000		37735		21527	27325	-4818	**×**	**×**
95	1000	16.11	13215	0.95	-2994	-3141	23207	***	***
	1500		19040		2832	2971	17382	**√**	**√**
	2000		24865		8657	9083	11557	**×**	**√**
	2500		30691		14482	15195	5732	**×**	**√**
	3000		36516		20307	21307	-94	**×**	**×**

SVR, sustained virologic response; QALY, quality-adjusted life year; ICER, Incremental cost-effectiveness ratio; NB, net monetary benefit; GDP, gross domestic product; GDP per capita of 2014 was $7070 (1 USD = 6.6594 CNY), 3×GDP per capita = $21209;

***: all-oral regimen dominates PegIFN regimen at present under specific assumption.

**Fig 4:**
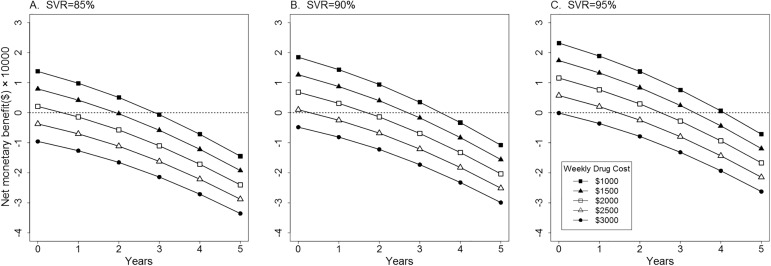
Impact of SVR rate and weekly drug cost on cost-effectiveness of delaying treatment for all-oral regimen in the next five years. SVR rate ranged from 85% to 95% and weekly drug cost ranged from $1000 to $3000. Immediate treatment of PegIFN regimen worked as the comparator strategy and net monetary benefit was calculated. SVR, sustained virologic response; PegIFN, peginterferon α-2a.

Moreover, patients could wait until all-oral regimen was available in China if it had relatively lower weekly drug cost. Under the assumed SVR rate, delaying treatment for all-oral regimen in the next two years from now on appeared to be cost-effective when weekly drug cost was less than $1500 (Fig 4). But if weekly drug cost achieved more than $2500 per week, initiating PegIFN regimen at present would be optimal.

**Fig 5:**
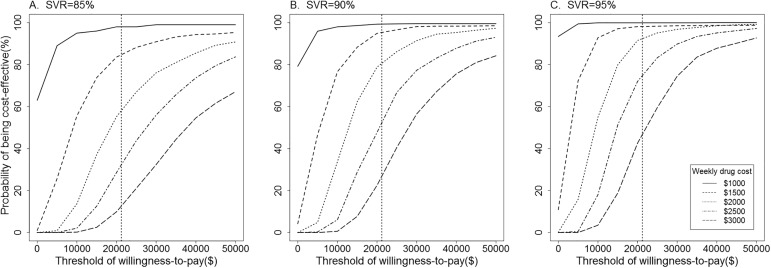
Probabilistic sensitivity analysis on cost-effectiveness of immediate treatment for all-oral regimen versus PegIFN regimen at present under assumed SVR rate and weekly drug cost. SVR rate ranged from 85% to 95% and weekly drug cost ranged from $1000 to $3000. SVR, sustained virologic response; PegIFN, peginterferon.

Furthermore, results of probabilistic sensitivity analysis were presented in Fig 5. At a willingness-to-pay threshold of $21209, initiating all-oral regimen at present was more than 95% likely to be cost-effective compared with PegIFN regimen if weekly drug cost was less than $1000. This probability decreased as weekly drug cost increased. When this cost reached $3000, the probability of all-oral regimen being cost-effective was less than 50%, on contrary, initiating PegIFN regimen at present was the optimal strategy for patients in China.

## Discussion

The revolution of novel regimens is being realized at a rapid pace in western counties, yet they have not been listed in China at present. Considering the large population of patients infected with HCV in China, the demand for these new drugs is exponentially increasing over time. Particularly, all-oral regimens are associated with excellent efficacy and safety protocols. In this study, the cost-effectiveness of initiating all-oral regimen at present or delaying treatment for all-oral regimen in the next five years for Chinese patients was assessed.

The results indicate that an all-oral regimen greatly improved clinical and economic effectiveness, which is also supported by previous cost-effectiveness analyses of all-oral regimens [45, 46]. From a consumer perspective, immediate treatment with all-oral regimen under assumed weekly drug cost and the SVR rate appeared to be more cost-effective compared with PegIFN regimen. In a one-way sensitivity analysis, an all-oral regimen also remained cost-effective. However, it is important to note that the higher cost of all-oral regimen could substantially increase short-term expenditure for public health and private payers. Nevertheless, if drug cost of all-oral regimen was less than $1,000 per week, it could actually be cost-saving. Furthermore, if the weekly drug cost of an all-oral regimen was less than $2,000 and the SVR rate was more than 90%, initiating an all-oral regimen at present was more than 80% likely to be cost-effective compared with the PegIFN regimen.

Due to the potential side effects and redundant treatment duration, several patients may not accept treatment at present. In this case, patients may delay treatment and wait until the all-oral regimen is listed in China with a lower price. Therefore, another important, though controversial, issue is whether delaying treatment for an all-oral regimen in the next five years is cost-effective. In our analysis, this strategy consistently appeared to be cost-effective for patients diagnosed with F1 or F2 fibrosis in all age groups. But for patients diagnosed with F0 fibrosis, they were expected to experience less advanced disease progression than those diagnosed with F1-F4 fibrosis. Therefore, treatment with PegIFN regimen for them also gained higher QALYs, resulting in a reduction in the incremental effect compared with an all-oral regimen, almost always leading to a negative NB. Accordingly, immediate treatment with PegIFN regimen may be optimal even if we expect that an all-oral regimen will soon become available in China. Because of the great impact of fibrosis stages on cost-effectiveness, HCV screening and fibrosis assessment need to be promoted for individual patients at varying ages. In the future, individualized medical treatment and economic evaluation may become increasingly common. Moreover, the price and SVR rate of an all-oral regimen is not yet known in China, and even more novel regimens are likely to enter the market along with great competition of drug price. For that reason, weekly drug cost and SVR rates were varied in the sensitivity analysis.

Since there is no comprehensive cost-effectiveness analysis of novel regimen for HCV in China, our study worked as a reference for economic evaluation for the near future of China. The ideas and findings from our study are supported by those from previous cost-effectiveness analyses that also investigated the decision of delaying treatment. Leidner A.J. et al [47] investigated the cost-effectiveness for treatment at various fibrosis stages against delaying treatment until the subsequent fibrosis stages. In their study, immediate treatment of HCV-infected patients with moderate and advanced fibrosis (F2-F4) appears to be cost-effective. In some scenarios, earlier treatment can be a cost-effective use of resources. Additionally, Deuffic-Burban S. et al [48] investigated the cost-effectiveness for immediate treatment with interferon-based regimens in contrast with delaying treatment for all-oral regimens. They found that awaiting IFN-free regimens and then treating all patients regardless of fibrosis stage proves to be an even more efficient strategy (with the exception of patients at F4 fibrosis stage). In the near future, with the opening up of the China market for novel regimens of HCV, more analyses need to be carried out in order to evaluate the balance between health benefit and expenditures, leading to overall better decisions for clinicians and patients.

There are several limitations and concerns for our study. First, the analysis only included treatment-naive patients with genotype 1. For future research, an analysis involving a more specific patient population would be necessary for China. Because of the limited data, adherence was not considered within the simulation. Second, the symptoms of patients infected with HCV may alter over time. More recent and comprehensive cross-sectional studies need to be conducted to collect more accurate information about these changing characteristics. In particular, METAVIR fibrosis scores should be promoted in China to obtain an accurate assessment of fibrosis stages. Owing to the lack of relevant studies done in China, the annual transition probabilities in the simulation were mainly based on the results from a US setting. Furthermore, effectiveness measurements were mainly described as QALYs that were based on the utilities. Utilities of several disease states in our study were derived from the studies on Hepatitis B in the Chinese population with the assumption that the quality of life was similar between these two diseases. All of these assumptions may lead to imprecision and bias. On account of that, more specific cohort studies need to be conducted to obtain more accurate data of utilities and annual costs for Chinese patients. In addition, the costs associated with the management of adverse events were not included from a conservative point of view, resulting in an under-estimation of cost-effectiveness even though a minimal impact was made. In future studies, real-world and clinical data for Chinese patients should be of particular importance.

In summary, an all-oral regimen is associated with acceptable long-term health and economic benefits in treatment-naive patients infected with HCV genotype 1. In particular, delaying treatment for the all-oral regimen may be a suitable choice if this regimen would become available at lower prices in the future. Moreover, patients with varying ages or fibrosis stages may also lead to mixed results. For those diagnosed with F1 or F2 fibrosis, delaying treatment for an all-oral regimen was recommended in the next five years under the base-case assumption. In general, an appropriate strategy needs to be chosen based on an assortment of factors to maximize both health and economic benefits.

## Supporting information

S1 Table Annual transition probabilities. F0–F4, Metavir fibrosis score; DC, decompensated cirrhosis; HCC, hepatocellular carcinoma; LT, liver transplant; PLT, post-liver transplant; LD, liver-related death; SVR, sustained virologic response; F4 SVR, patients diagnosed at F4 fibrosis stage achieved SVR.
(DOCX)

S2 Table Treatment costs and annual health state costs. F0–F4, METAVIR liver fibrosis scores. DC, decompensated cirrhosis; HCC, hepatocellular carcinoma; LT, liver transplant; PLT, post-liver transplant. All costs were converted to USD using official exchange rates as of 2016 (1 USD = 6.6594 CNY) and were inflated to 2016 prices using China Consumer Price Index (CPI).
(DOCX)

S3 Table Utilities of different health states. F0–F4, METAVIR liver fibrosis scores. DC, decompensated cirrhosis; HCC, hepatocellular carcinoma; LT, liver transplant; PLT, post-liver transplant; SVR, sustained virologic response.
(DOCX)

S1 Fig Investigate the between-model consistency by comparing lifetime costs and QALYs. The dotted line was fitted according to the exact data from the literature; The solid line was fitted according to the simulated data from the model (*R*^*2*^ = 0.997 for QALYs; *R*^*2*^ = 0.979 for costs). The comparison of regression coefficients of these two lines illustrated that the simulated data from the model was consistent with that from the published literature (*P* = 0.128 and *P* = 0.161, respectively).
(TIF)

S2 Fig One-way sensitivity analysis for all-oral regimen versus PegIFN regimen at present. This analysis was conducted with fixed weekly drug cost of $2000 and SVR rate of 90% for all-oral regimen. Only top fifteen sensitive parameters were included in the diagram and incremental cost-effectiveness ratio was calculated. SVR, sustained virologic response; PegIFN, peginterferon α-2a; F0-F4, Metavir fibrosis scores; ICER, incremental cost-effectiveness ratio.
(TIF)
