# Supplementary material for: Estimating cost-effectiveness associated with all-oral regimen for chronic hepatitis C in China
Source: PLoS One. 2017 Apr 5;12(4):e0175189. doi: 10.1371/journal.pone.0175189 (PMC5381915; doi:10.1371/journal.pone.0175189)
Supplement: S1 Table — F0–F4, Metavir fibrosis score; DC, decompensated cirrhosis; HCC, hepatocellular carcinoma; LT, liver transplant; PLT, post-liver transplant; LD, liver-related death; SVR, sustained virologic response; F4 SVR, patients diagnosed at F4 fibrosis stage achieved SVR. (DOCX) [file pone.0175189.s001.docx]

**S1 Table. Annual transition probabilities.**

|  | Base-Case Value (Range) | Distribution (α, β) | Ref. |
| --- | --- | --- | --- |
| Natural history of chronic hepatitis C | | | |
| F0-F1 | 0.117 (0.105-0.129) | Beta (85, 639) | 18 |
| F1-F2 | 0.085 (0.077-0.094) | Beta (88, 945) |  |
| F2-F3 | 0.120 (0.108-0.132) | Beta (84, 619) |  |
| F3-F4 | 0.116 (0.104-0.128) | Beta (85, 646) |  |
| F3-DC | 0.012 (0.011-0.013) | Beta (95, 7811) | 19, 20 |
| F3-HCC | 0.011 (0.010-0.012) | Beta (95, 8539) |  |
| F4-DC | 0.039 (0.031-0.047) | Beta (92, 2273) |  |
| F4-HCC | 0.024 (0.019-0.029) | Beta (94, 3811) |  |
| DC-HCC | 0.068 (0.054-0.082) | Beta (89, 1226) | 21, 22 |
| DC-LT | 0.023 (0.018-0.028) | Beta (94, 3985) |  |
| DC-LD | 0.104 (0.083-0.125) | Beta (86, 740) | 23-25 |
| HCC-LT | 0.040 (0.032-0.048) | Beta (92, 2212) | 22 |
| HCC-LD | 0.520 (0.416-0.624) | Beta (46, 42) | 23-25 |
| LT-LD | 0.194 (0.155-0.233) | Beta (77, 321) | 26 |
| PLT-LD | 0.049 (0.039-0.059) | Beta (91, 1772) |  |
| Achieve SVR | | | |
| F4 SVR-DC | 0.003 (0.002-0.004) | Beta (96, 31821) | 19 |
| F4 SVR-HCC | 0.006 (0.005-0.007) | Beta (95, 15814) |  |

F0–F4, Metavir fibrosis score; DC, decompensated cirrhosis; HCC, hepatocellular carcinoma; LT, liver transplant; PLT, post-liver transplant; LD, liver-related death; SVR, sustained virologic response; F4 SVR, patients diagnosed at F4 fibrosis stage achieved SVR.
