# Supplementary material for: Estimating cost-effectiveness associated with all-oral regimen for chronic hepatitis C in China
Source: PLoS One. 2017 Apr 5;12(4):e0175189. doi: 10.1371/journal.pone.0175189 (PMC5381915; doi:10.1371/journal.pone.0175189)
Supplement: S2 Table — F0–F4, METAVIR liver fibrosis scores. DC, decompensated cirrhosis; HCC, hepatocellular carcinoma; LT, liver transplant; PLT, post-liver transplant. All costs were converted to USD using official exchange rates as of 2016 (1 USD = 6.6594 CNY) and were inflated to 2016 prices using China Consumer Price Index (CPI). (DOCX) [file pone.0175189.s002.docx]

**S2 Table. Treatment costs and annual health state costs.**

|  | Base-case Value (Range, $) | Distribution (α, β) | Ref. |
| --- | --- | --- | --- |
| Weekly drug cost | | | |
| All-oral regimen | Wide Range (1000-3000) | Gamma (96.04, 10.41-31.24) | Expert opinion |
| Peginterferon α-2a plus Ribavirin | 174 (±20%) | Gamma (96.04, 1.82) |  |
| Weekly monitoring cost | 23 (±20%) | Gamma (96.04, 0.24) |  |
| Annual health states cost | | | |
| F0-F3 | 1202 (±20%) | Gamma (96.04, 12.52) | 30 |
| F4 | 1245 (±20%) | Gamma (96.04, 12.96) |  |
| DC | 3236 (±20%) | Gamma (96.04, 33.69) | 31 |
| HCC | 5772 (±20%) | Gamma (96.04, 60.10) | 32 |
| LT | 50963 (±20%) | Gamma (96.04, 530.64) | 33, 34 |
| PLT | 8493 (±20%) | Gamma (96.04, 88.43) |  |

F0–F4, METAVIR liver fibrosis scores. DC, decompensated cirrhosis; HCC, hepatocellular carcinoma; LT, liver transplant; PLT, post-liver transplant. All costs were converted to USD using official exchange rates as of 2016 (1 USD=6.6594 CNY) and were inflated to 2016 prices using China Consumer Price Index (CPI).
