# Supplementary material for: Estimating cost-effectiveness associated with all-oral regimen for chronic hepatitis C in China
Source: PLoS One. 2017 Apr 5;12(4):e0175189. doi: 10.1371/journal.pone.0175189 (PMC5381915; doi:10.1371/journal.pone.0175189)
Supplement: S3 Table — F0–F4, METAVIR liver fibrosis scores. DC, decompensated cirrhosis; HCC, hepatocellular carcinoma; LT, liver transplant; PLT, post-liver transplant; SVR, sustained virologic response. (DOCX) [file pone.0175189.s003.docx]

**S3 Table. Utilities of different health states.**

|  | Base-case Value (Range) | Distribution (α, β) | Ref. |
| --- | --- | --- | --- |
| Utilities with disease stages | | | |
| F0-F3 | 0.790 (0.632-0.948) | Beta (19.4, 5.2) | 37 |
| F4 | 0.748 (0.598-0.898) | Beta (23.5, 7.9) |  |
| DC | 0.672 (0.538-0.806) | Beta (30.8, 15.0) |  |
| HCC | 0.610 (0.488-0.732) | Beta (36.8, 23.6) |  |
| LT | 0.650 (0.520-0.780) | Beta (33.0, 17.8) |  |
| PLT | 0.709 (0.567-0.851) | Beta (27.2, 11.2) |  |
| Utilities after achieving SVR | | | |
| F0-F3 | 0.840 (0.672-1.000) | Beta (15.3, 2.9) | 38 |
| F4 | 0.799 (0.639-0.959) | Beta (18.5, 4.7) |  |
| Utilities during interferon-based treatment | | | |
| F0-F3 | 0.675 (0.540-0.810) | Beta (30.6, 14.7) | 39 |
| F4 | 0.639 (0.511-0.767) | Beta (34.1, 19.3) |  |
| Utilities during all-oral treatment | | | |
| F0-F3 | 0.826 (0.660-0.991) | Beta (15.9, 3.4) | 39 |
| F4 | 0.782 (0.625-0.938) | Beta (20.2, 5.6) |  |

F0–F4, METAVIR liver fibrosis scores. DC, decompensated cirrhosis; HCC, hepatocellular carcinoma; LT, liver transplant; PLT, post-liver transplant; SVR, sustained virologic response.
