# Supplementary figures and images for: Estimating cost-effectiveness associated with all-oral regimen for chronic hepatitis C in China
Source: PLoS One. 2017 Apr 5;12(4):e0175189. doi: 10.1371/journal.pone.0175189 (PMC5381915; doi:10.1371/journal.pone.0175189)

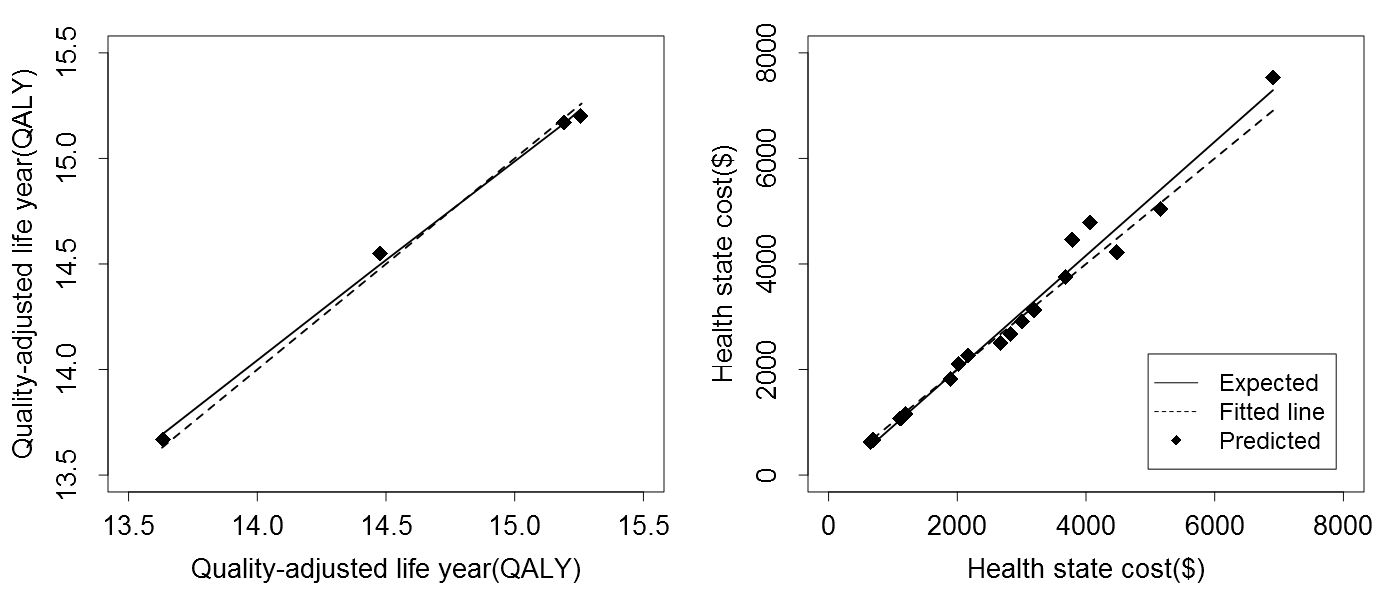

Supplement: S1 Fig — The dotted line was fitted according to the exact data from the literature; The solid line was fitted according to the simulated data from the model (R2 = 0.997 for QALYs; R2 = 0.979 for costs). The comparison of regression coefficients of these two lines illustrated that the simulated data from the model was consistent with that from the published literature (P = 0.128 and P = 0.161, respectively). (TIF) [file pone.0175189.s004.tif]

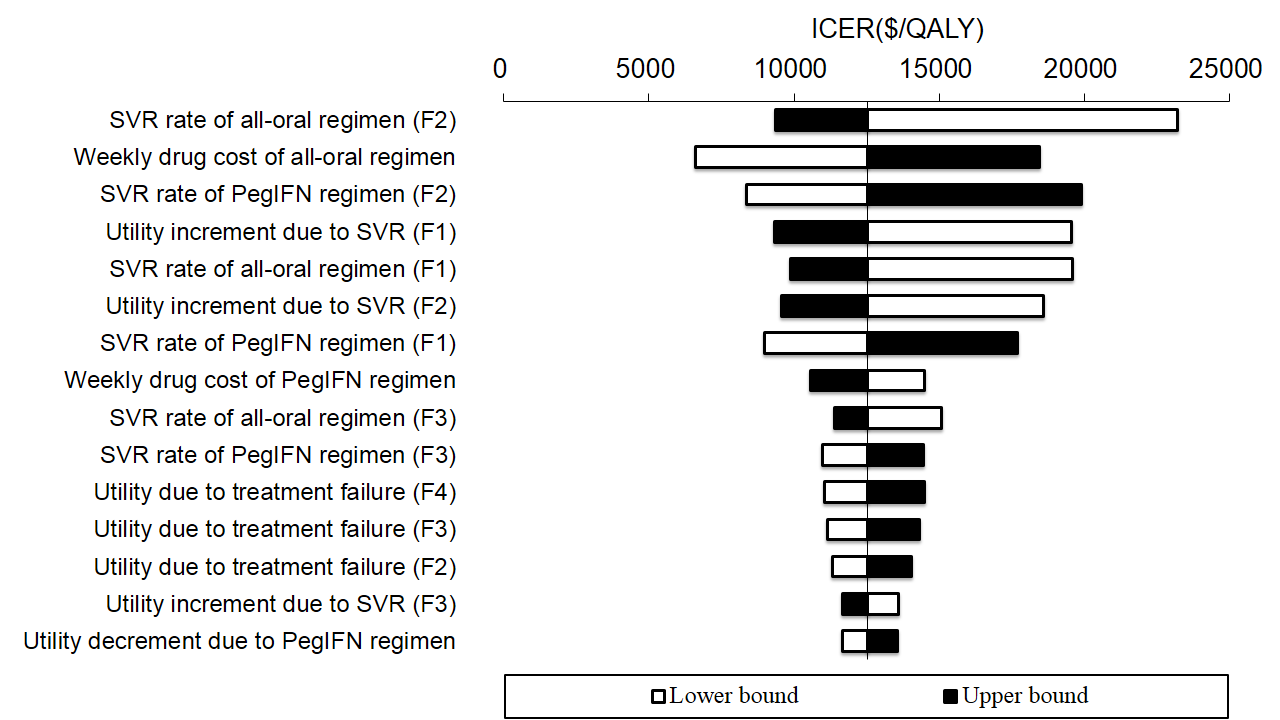

Supplement: S2 Fig — This analysis was conducted with fixed weekly drug cost of $2000 and SVR rate of 90% for all-oral regimen. Only top fifteen sensitive parameters were included in the diagram and incremental cost-effectiveness ratio was calculated. SVR, sustained virologic response; PegIFN, peginterferon α-2a; F0-F4, Metavir fibrosis scores; ICER, incremental cost-effectiveness ratio. (TIF) [file pone.0175189.s005.tif]
